# HDAC6抑制剂ACY-738通过乙酰化P53诱导弥漫大B细胞淋巴瘤细胞凋亡和自噬

**DOI:** 10.3760/cma.j.cn121090-20240826-00324

**Published:** 2025-05

**Authors:** 佩洁 蒋, 金宜 刘, 官翠 杨, 佳润 李, 小龙 田, 世杰 杨, 锦 魏, 曦 张

**Affiliations:** 1 川北医学院附属医院血液内科，南充 637000 Department of Hematology, Affiliated Hospital of North Sichuan Medical College, Nanchong 637000, China; 2 陆军军医大学第二附属医院血液病医学中心，血液病与微环境重庆市重点实验室，重庆 400037 Chongqing Key Laboratory of Hematology and Microenvironment, Medical Center of Hematology, Second Affiliated Hospital, Army Medical University, Chongqing 400037, China

**Keywords:** 组蛋白去乙酰化酶6, 淋巴瘤，大B细胞，弥漫性, 凋亡, 自噬, HDAC6, Lymphoma, large B-cell, diffuse, Apoptosis, Autophagy

## Abstract

**目的:**

初步探讨组蛋白去乙酰化酶6（HDAC6）抑制剂ACY-738在弥漫大B细胞淋巴瘤（DLBCL）中的抗肿瘤作用及其机制。

**方法:**

生信分析HDAC6在不同肿瘤及DLBCL中的表达。不同浓度ACY-738处理DLBCL细胞后，CCK-8检测细胞活力；EdU检测细胞DNA合成能力；软琼脂实验检测细胞克隆形成；荧光显微镜检测细胞内活性氧（ROS）的水平；透射电镜观察细胞形态的变化；流式细胞术检测线粒体ROS和细胞凋亡水平；蛋白质印迹法（Western blotting）检测细胞凋亡相关蛋白及自噬相关蛋白的表达水平。

**结果:**

HDAC6在DLBCL中高表达（*P*<0.05）。ACY-738抑制DLBCL细胞增殖、DNA合成及克隆形成，且呈剂量依赖性（*P*<0.05）。ACY-738处理后DLBCL细胞内ROS水平及线粒体ROS水平增加，且呈剂量依赖性（*P*<0.05）。电镜观察细胞形态，发现ACY-738处理后，细胞线粒体肿胀破裂，线粒体嵴减少或消失，出现自噬溶酶体，并观察到细胞凋亡形态；Western blotting结果显示ACY-738处理后，凋亡相关蛋白BCL-2表达下调，Cleaved-PARP、Cleaved caspase-3、BAX表达上调（*P*<0.05），自噬相关蛋白Atg7、Atg3、LC3B、P62表达下调，乙酰化P53蛋白表达上调（*P*<0.05）。

**结论:**

HDAC6抑制剂ACY-738通过乙酰化P53诱导线粒体依赖性细胞凋亡和自噬，从而抑制DLBCL细胞生长。

## Abstract (en)

**Objective:**

To investigate the anti-tumor effect of the Histone Deacetylase 6（HDAC6）inhibitor ACY-738 and its underlying mechanisms in Diffuse Large B-cell Lymphoma（DLBCL）.

**Methods:**

The expression of HDAC6 in various tumors and DLBCL was analyzed using bioinformatics. DLBCL cells were treated with different concentrations of ACY-738. Cell viability, DNA synthesis, and clone formation were assessed by CCK-8 assay, EdU assay, and soft agar assay, respectively. Intracellular reactive oxygen species（ROS）levels were detected by fluorescence microscopy. Morphological changes in cells were observed using transmission electron microscopy（TEM）. Mitochondrial ROS levels and apoptosis were measured by flow cytometry. The expression levels of apoptosis-related and autophagy-related proteins were detected by Western blotting.

**Results:**

HDAC6 was highly expressed in DLBCL（*P*<0.05）. ACY-738 inhibited the proliferation, DNA synthesis, and colony formation of DLBCL cells in a dose-dependent manner（*P*<0.05）. Treatment with ACY-738 increased intracellular and mitochondrial ROS levels in DLBCL cells in a dose-dependent manner（*P*<0.05）. TEM revealed that after ACY-738 treatment, mitochondria in cells were swollen and ruptured, mitochondrial cristae were reduced or absent, autolysosomes appeared, and features characteristic of apoptosis were observed. Western blotting showed that after ACY-738 treatment, the expression of the anti-apoptotic protein BCL-2 was downregulated, while the expression of Cleaved-PARP, Cleaved caspase-3, and BAX was upregulated（*P*<0.05）. The expression of autophagy-related proteins Atg7, Atg3, LC3B, and P62 was downregulated, and the expression of acetylated P53 protein was upregulated（*P*<0.05）.

**Conclusion:**

The HDAC6 inhibitor ACY-738 induces mitochondria-dependent apoptosis and autophagy in DLBCL cells by acetylating P53, thereby inhibiting DLBCL cell proliferation.

弥漫大B细胞淋巴瘤（DLBCL）是一种侵袭性非霍奇金淋巴瘤，占所有淋巴瘤的30％～40％，亦是国内最常见的淋巴瘤类型[1]–[2]。目前DLBCL常用治疗策略为R-CHOP（利妥昔单抗+环磷酰胺+多柔比星+长春新碱+泼尼松）方案，但由于其高侵袭性与异质性等特点，致使约30％患者仍存在难治或复发情况[3]–[4]。因此，探索DLBCL新的治疗靶点及药物，对于降低DLBCL相关死亡率和提高总体生存率至关重要。

目前，已有多种组蛋白去乙酰化酶（HDAC）抑制剂，如伏立诺他、罗米地辛、西达本胺被批准用于治疗T细胞淋巴瘤[5]–[9]，但在B细胞淋巴瘤中仍处于探索阶段，研究表明，HDAC抑制剂恩替司他通过降低BCL-XL水平进而诱导B细胞淋巴瘤的半胱天冬酶依赖性凋亡[10]–[11]。HDAC抑制剂通过多种机制抑制肿瘤细胞增殖，包括上调死亡受体和促凋亡蛋白、诱导氧化应激、破坏细胞周期检查点和DNA修复等[12]。研究显示，HDAC6作为现有18种HDAC家族中的Ⅱb类成员[13]，具有成为治疗恶性肿瘤靶点的潜力，针对淋巴瘤所设计的HDAC6抑制剂也已处于临床试验中[14]。本实验将以DLBCL细胞为研究对象，探讨HDAC6抑制剂ACY-738对DLBCL的治疗效果及其机制，为HDAC6抑制剂在DLBCL临床治疗中的应用提供实验依据。

## 材料与方法

一、材料

1. 细胞：DLBCL细胞系SU-DHL-2、SU-DHL-6、OCI-LY1、OCI-LY3、Toledo、Pfeiffer购自广州吉尼欧生物科技有限公司。

2. 试剂与仪器：胎牛血清（FBS）和RPMI 1640细胞培养基购自美国Gibco公司。IMDM细胞培养基购自以色列BI公司。二甲基亚砜（DMSO）购自美国Sigma公司。HDAC6抑制剂ACY-738及红色线粒体超氧化物荧光探针（Mito SOX Red）购自美国MCE公司。CCK-8购自日本同仁公司。Agarose购自瑞士Lonza公司。3-（4,5-二甲基-2-噻唑基）-2,5-二苯基四氮唑溴盐（MTT）购自美国Invitrogen公司。HDAC6腺病毒试剂购自中国上海汉恒生物公司。Annexin V-APC/7aad细胞凋亡检测试剂盒购自美国BD公司。EdU-594细胞增殖检测试剂盒、活性氧（ROS）、RIPA裂解液、蛋白酶抑制剂混合物、BCA蛋白浓度测定试剂盒、蛋白质印迹法（Western blotting）一抗稀释液和二抗稀释液、封闭液均购自中国碧云天生物技术有限公司。GAPDH、β-actin、Cleaved-PARP、Cleaved caspase-3、BCL-2、BAX、Acetyl-P53、Atg7、P62、Atg3、LC3B、HDAC6抗体及辣根过氧化物酶山羊抗兔二抗均购自美国CST公司。ECL发光试剂盒购自美国Millipore公司。多功能酶标仪购自美国Thermo Fisher公司。正置荧光显微镜购自日本Olympus公司。流式细胞仪购自美国Beckman Coulter公司。电泳仪、Chemi Doc XRS+Imager高灵敏度化学发光成像系统购自美国Bio-Rad公司。

二、方法

1. ACY-738的配制：ACY-738用DMSO配制成10 mmol/L母液，用1×PBS稀释成5、10、20 µmol/L工作液，−20 °C分装保存。

2. 细胞培养与分组：OCI-LY1、OCI-LY3细胞采用含20％ FBS的IMDM培养基，SU-DHL-6、SU-DHL-2、Toledo、Pfeiffer细胞采用含10％ FBS的1640培养基，在37 °C，5％ CO_2_细胞培养箱中培养细胞，取对数生长期细胞用于实验。后续实验根据ACY-738浓度分为对照组（0 µmol/L）和实验组（5、10、20 µmol/L）。

3. HDAC6在肿瘤中的表达分析：采用GEPIA（http://gepia.cancer-pku.cn/index.html）数据库分析HDAC6在不同肿瘤及DLBCL中的表达。采用GEO数据集GSE56315分析HDAC6在DLBCL组织和正常组织中的表达。

4. CCK8检测：细胞以1×10^4^个/孔接种于96孔板。加入不同浓度的ACY-738（0、0.03、0.1、0.3、1、3、10、30、100、300 µmol/L）作用24 h，每组设置3个复孔，每孔加入CCK-8试剂10 µl，37 °C继续培养2 h，使用酶标仪检测450 nm处的吸光度（*A*）值，并按照以下公式计算细胞抑制率。

细胞抑制率（％）＝（1−*A*_实验组_/*A*_对照组_）×100％

5. EdU检测：细胞以1×10^6^个/孔接种于6孔板，加入4种浓度的ACY-738（0、5、10、20 µmol/L）作用24 h后加入EdU工作液孵育2 h，随后用4％多聚甲醛固定细胞15 min，PBS洗3次后加入通透液孵育15 min，随后加入EdU反应液避光孵育30 min，PBS洗涤3次后，加入Hoechst 33342染色30 min，荧光显微镜下观察，随机选取3个视野拍照，计算阳性细胞率。

6. 软琼脂克隆形成实验：用培养基将3.5％ Agar胶稀释为0.7％的下层胶，以2 ml每孔的量加入6孔板中，4 °C静置15 min凝固。分别对OCI-LY1和SU-DHL-6细胞进行计数（3 000/孔），将细胞重悬于0.35％ Agar胶中，加入4种浓度的ACY-738（0、5、10、20 µmol/L），1.5 ml每孔，4 °C静置1～2 h凝固后，放入37 °C培养箱。2～3周后加入200 µl MTT，37 °C，5％ CO_2_孵育30 min后观察拍照，并对形成克隆进行计数。

7. ROS检测：细胞以1×10^6^个/孔接种于6孔板，加入4种浓度的ACY-738作用24 h后，收集细胞，PBS洗1次，加入终浓度为1 µmol/L的DCFH-DA工作液500 µl，室温避光孵育30 min后，PBS洗1次，加入Hoechst 33342染色30 min，荧光显微镜下观察，随机选取3个视野拍照，计算阳性细胞率。

8. 线粒体ROS检测：细胞以1×10^6^个/孔接种于6孔板，加入4种浓度的ACY-738作用24 h后，收集细胞，PBS洗1次，加入终浓度为1 µmol/L的Mito Sox Red工作液500 µl，室温避光孵育30 min后用流式细胞仪检测线粒体ROS变化。

9. 透射电镜检测：细胞以1×10^6^个/孔接种于6孔板，加入4种浓度的ACY-738作用24 h后，收集细胞，PBS洗1次，加入1 ml电镜固定液室温固定2 h，再转移至4 °C保存，后由武汉塞维尔生物科技有限公司完成后续检测。

10. 细胞凋亡检测：细胞以1×10^6^个/孔接种于6孔板，加入4种浓度的ACY-738作用24 h后，收集细胞，PBS洗1次，加入Annexin V-APC及7aad染料各2.5 µl，室温避光孵育30 min后用流式细胞仪检测细胞凋亡情况。

11. HDAC6敲低细胞株构建：细胞以3×10^5^个孔接种于24孔板，加入2 µl shHDAC6腺病毒母液，吹打混匀5～10次，室温静置15 min后补300 µl培养基，在37 °C，5％ CO_2_细胞培养箱中培养2 d，通过荧光显微镜观察感染率。

12. 蛋白提取及Western blotting检测：细胞以1×10^6^个/孔接种于6孔板，加入4种浓度的ACY-738作用24 h后，收集细胞，加入蛋白裂解液冰上裂解30 min，13 400×*g*、4°C离心10 min后取上清。使用BCA蛋白定量试剂盒检测蛋白浓度并定量。加入蛋白上样缓冲液，100 °C金属浴加热10 min。电泳后将分离的蛋白转膜至PVDF膜上，快速封闭液封闭15 min，孵育一抗4 °C过夜，Cleaved-PARP、Cleaved caspase-3、BAX、BCL-2、Acetyl-P53、Atg7、Atg3、P62、LC3B、HDAC6稀释比例为1∶1 000，GAPDH、β-actin稀释比例均为1：3 000。二抗室温孵育2 h。配制显影液，化学发光仪显影并拍照。使用Image J软件检测蛋白条带灰度值。目的蛋白相对表达量以目的蛋白与内参灰度值的比值表示。

三、统计学处理

采用GraphPad Prism 9.0软件进行统计分析与作图。数据以*x*±*s*表示，两组间比较采用Student's *t*检验，多组间比较采用单因素方差分析，每次实验至少重复3次，以*P*<0.05为差异有统计学意义。

## 结果

一、生信分析HDAC6在DLBCL中高表达

生物信息学分析表明，HDAC6在DLBCL中高表达（*P*<0.05，图1A、B）。Western blotting结果表明，与对照相比，HDAC6高表达于DLBCL细胞，具体到不同的细胞系中，OCI-LY1（HDAC6/GAPDH：0.94±0.02）、SU-DHL-6（HDAC6/GAPDH：1.15±0.13）细胞差异均有统计学意义（均*P*<0.05，图1C）。提示HDAC6可能作为DLBCL的潜在治疗靶点。

**图1:**
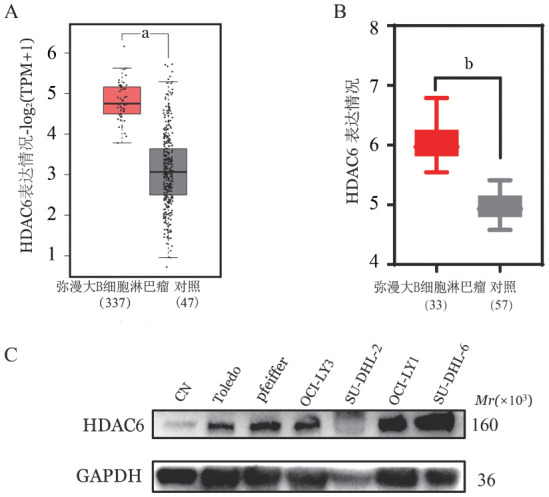
HDAC6在弥漫大B细胞淋巴瘤（DLBCL）中的表达 **A、B** 采用GEPIA或GEO数据集分析HDAC6在DLBCL组织及正常组织中的表达；**C** HDAC6在正常细胞对照组和DLBCL细胞的蛋白表达 **注** 与对照组（CN）比较，^a^*P*<0.05；^b^*P*<0.001

二、ACY-738抑制DLBCL细胞增殖

采用CCK-8实验检测ACY-738对DLBCL细胞增殖的影响。结果表明，与对照组相比，ACY-738作用24 h后SU-DHL-2、SU-DHL-6、OCI-LY1、OCI-LY3、Toledo、Pfeiffer细胞增殖能力明显受到抑制，且在OCI-LY1、SU-DHL-6细胞中呈现剂量依赖性，即浓度越高，细胞增殖抑制性越强（*P*<0.05）。因此，选用OCI-LY1、SU-DHL-6细胞进行后续实验。ACY-738作用24 h后通过对药物半数抑制浓度（IC_50_）进行拟合，OCI-LY1细胞IC_50_为7.33 µmol/L、SU-DHL-6细胞IC_50_为12.03 µmol/L。EdU结果表明，ACY-738给药组能够显著降低OCI-LY1、SU-DHL-6细胞的DNA合成能力（*P*<0.05，图2A-D）。软琼脂克隆结果表明，与对照组相比，ACY-738给药组能够显著抑制OCI-LY1、SU-DHL-6细胞克隆形成（*P*<0.05，图2E、F）。以上结果表明ACY-738可以抑制DLBCL细胞增殖。

**图2:**
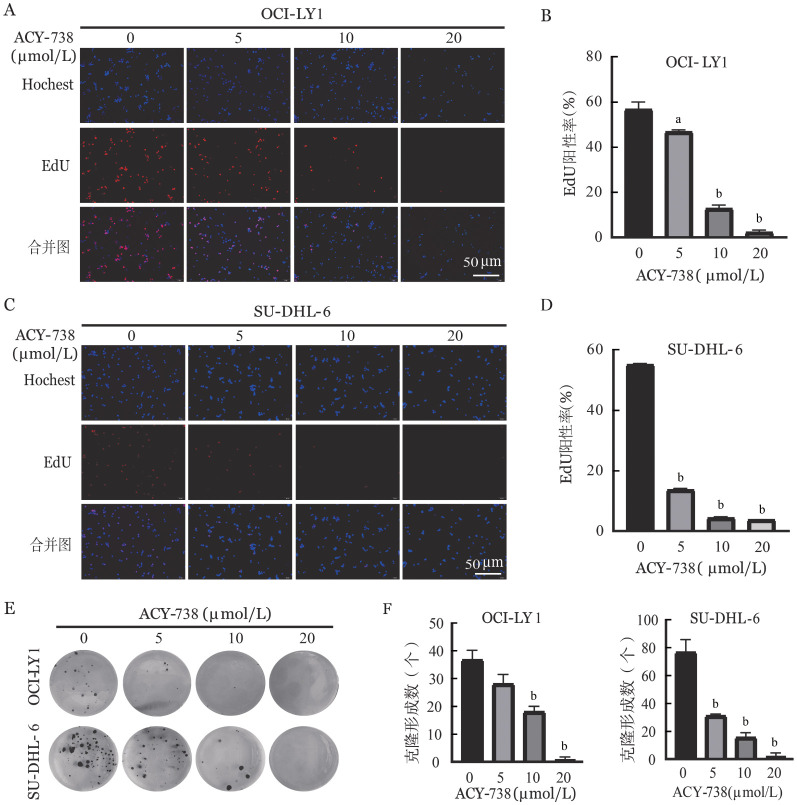
HDAC6抑制剂ACY-738抑制弥漫大B细胞淋巴瘤细胞（OCI-LY1、SU-DHL-6细胞）增殖 **A-D** ACY-738作用24 h后抑制OCI-LY1细胞和SU-DHL-6细胞DNA合成能力；**E、F** ACY-738作用24 h后抑制OCI-LY1和SU-DHL-6细胞克隆形成能力 **注** 与对照组（ACY-738 0 µmol/L）比较，^a^*P*<0.01；^b^*P*<0.001

三、ACY-738诱导DLBCL细胞线粒体损伤

通过荧光显微镜检测ACY-738作用后DLBCL细胞内ROS水平的变化。结果表明，与对照组相比，ACY-738处理后细胞ROS水平增加，且呈剂量依赖性（*P*<0.01，图3）。进一步通过流式细胞术检测ACY-738作用后DLBCL细胞线粒体ROS变化。结果表明，与对照组相比，ACY-738处理后线粒体ROS呈剂量依赖性增加（*P*<0.001，图3）。以上结果表明，ACY-738可以诱导线粒体氧化损伤，导致ROS产生及线粒体超氧化物增加。

**图3:**
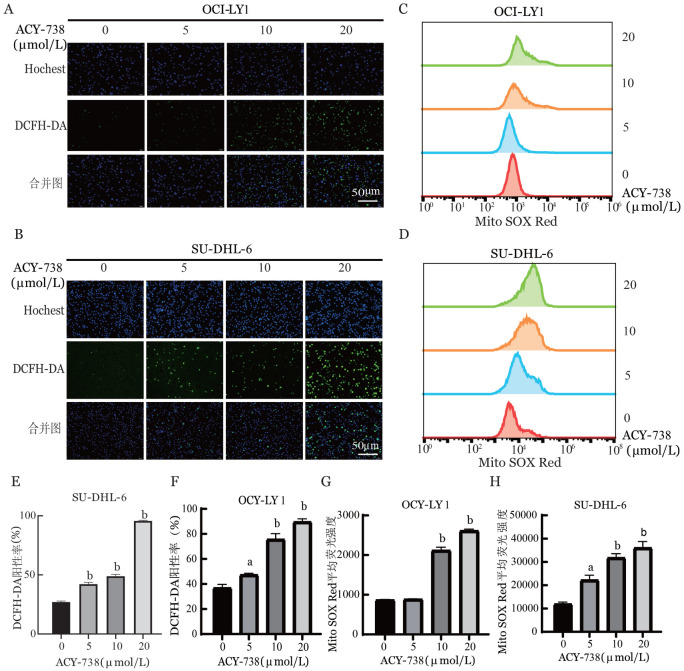
HDAC6抑制剂ACY-738诱导弥漫大B细胞淋巴瘤细胞（OCI-LY1、SU-DHL-6细胞）氧化应激 **A、B** ACY-738作用24 h后OCI-LY1细胞和SU-DHL-6细胞活性氧水平变化；**C、D** ACY-738作用24 h后OCI-LY1细胞和SU-DHL-6细胞线粒体活性氧水平变化；**E-H** 活性氧水平变化统计图 **注** 与对照组（ACY-738 0 µmol/L）比较，^a^*P*<0.01；^b^*P*<0.001

通过透射电镜对线粒体形态进行观察，与对照组相比，ACY-738处理后线粒体发生明显异常如肿胀、破裂，线粒体嵴断裂甚至消失。同时观察到ACY-738作用后DLBCL细胞中出现自噬溶酶体，此外，在高浓度组观察到明显的细胞凋亡形态。以上结果说明，ACY-738可能通过诱导线粒体损伤导致细胞发生凋亡和自噬。

四、ACY-738诱导DLBCL细胞凋亡

通过流式细胞术检测ACY-738作用后的细胞凋亡率。结果显示，与对照组相比，ACY-738处理后细胞凋亡率增加，且呈现剂量依赖性［ACY-738浓度为0、5、10、20 µmol/L时，OCI-LY1和SU-PHL-6细胞凋亡率分别为（9.57±0.33）％、（19.23±1.11）％、（47.30±0.36）％、（53.03±0.38）％；（11.28±0.75）％、（21.43±0.97）％、（29.43±1.10）％、（32.73±0.15）％］，其中以高浓度组最为显著，这与电镜结果一致（*P*<0.001，图4A）。Western blotting结果表明，ACY-738处理后，Cleaved-PARP、Cleaved caspase-3表达上调、抗凋亡蛋白BCL-2表达下调、促凋亡蛋白BAX表达上调（*P*<0.05，图4B）。以上结果表明，ACY-738通过调控BCL-2/BAX表达诱导细胞发生线粒体依赖的细胞凋亡。

**图4:**
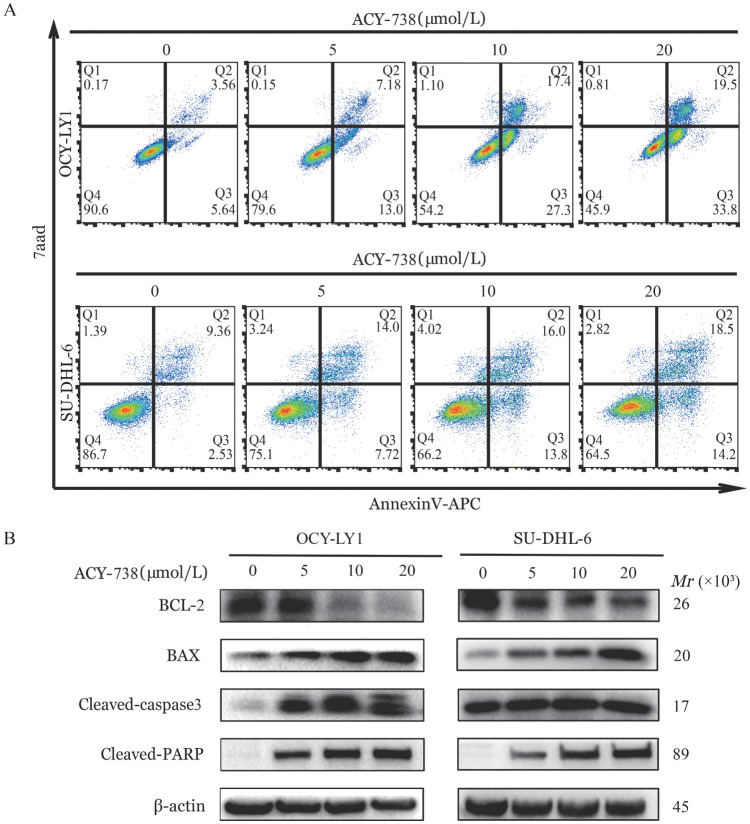
HDAC6抑制剂ACY-738诱导弥漫大B细胞淋巴瘤细胞（OCI-LY1、SU-DHL-6细胞）凋亡 **A** ACY-738作用24 h后OCI-LY1细胞和SU-DHL-6细胞凋亡率；**B** ACY-738作用24 h后OCI-LY1细胞和SU-DHL-6细胞凋亡相关蛋白的表达

五、ACY-738诱导DLBCL细胞自噬

Western blotting结果表明，ACY-738处理后，Atg3、Atg7表达下调，LC3B、P62表达显著下调（*P*<0.05，图5A）。说明ACY-738可以诱导DLBCL细胞发生自噬。进一步对其上游调控机制进行探索，研究发现，ACY-738处理后，P53乙酰化水平上调（*P*<0.05，图5B）。HDAC6敲低后，也得到了相似的结果（*P*<0.05，图5C）。以上提示ACY-738通过抑制HDAC6的表达，进而乙酰化激活P53，导致细胞发生凋亡和自噬。

**图5:**
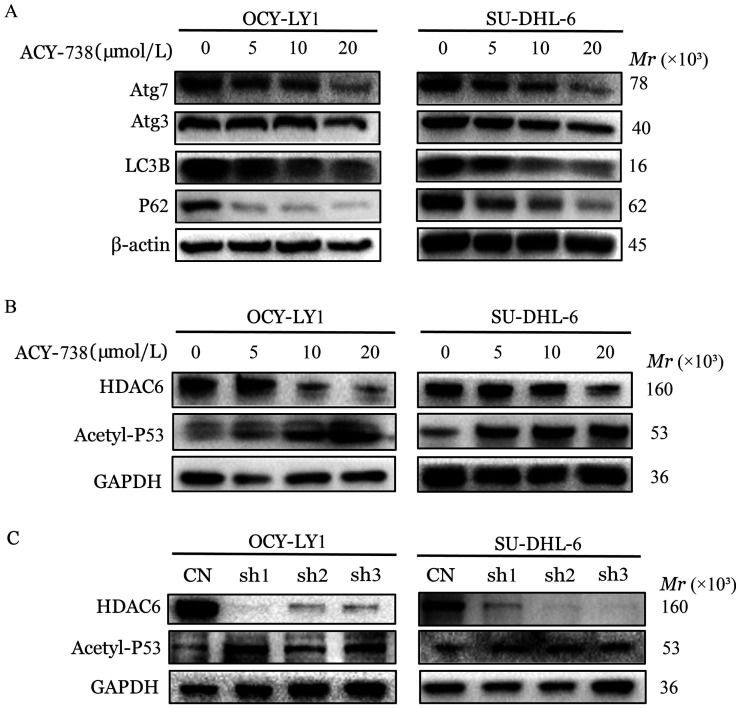
HDAC6抑制剂ACY-738诱导弥漫大B细胞淋巴瘤细胞（OCI-LY1、SU-DHL-6细胞）自噬 **A** ACY-738作用24h后OCI-LY1细胞和SU-DHL-6细胞自噬相关蛋白的表达；**B** ACY-738作用24 h后OCI-LY1细胞和SU-DHL-6细胞HDAC6及Acetyl-P53蛋白的表达；**C** HDAC6敲低后OCI-LY1细胞和SU-DHL-6细胞HDAC6及Acetyl-P53蛋白的表达 **注** CN：HDAC6未敲低细胞

## 讨论

HDAC6是HDAC家族成员之一，与多种肿瘤的发生发展密切相关[15]–[16]。抑制HDAC6的表达和功能是一种有前景的肿瘤治疗策略[17]–[18]。研究显示，ACY-738与伊布替尼联合用药可以延长慢性淋巴细胞白血病患者的总生存期，表明ACY-738在血液系统恶性肿瘤中具有一定的治疗前景[19]。本研究发现HDAC6在DLBCL中高表达，因此，我们推测HDAC6可能作为DLBCL的潜在治疗靶点。

本研究进一步发现ACY-738可诱导DLBCL细胞线粒体发生氧化损伤。此外，在电镜中观察到自噬溶酶体产生及明显的细胞凋亡形态，说明ACY-738抑制DLBCL细胞增殖可能与线粒体氧化损伤介导的凋亡和自噬有关。凋亡是一种程序性细胞死亡方式，其发生途径包括线粒体途径、内质网途径以及死亡受体途径。BCL-2是线粒体途径的重要凋亡调控因子，当线粒体发生氧化损伤时，抗凋亡蛋白BCL-2活性受到抑制，促凋亡蛋白BAX活性增强，从而释放凋亡因子，激活caspase-3，进一步激活效应PARP，启动caspase级联反应，最终导致细胞凋亡发生[20]–[21]。本研究发现，ACY-738处理后细胞凋亡率增加，BCL-2蛋白表达下调，BAX、Cleaved caspse-3、Cleaved-PARP蛋白表达上调。提示ACY-738可以通过线粒体依赖途径诱导DLBCL细胞发生凋亡。

自噬是维持细胞内环境稳态的生理过程，其中LC3是公认的自噬标志物，具有控制自噬膜形成和自噬体与溶酶体融合的功能。自噬体和溶酶体融合后，外膜上的LC3-Ⅱ被切割，产生LC3-Ⅰ循环利用；内膜上的LC3-Ⅱ被溶酶体酶降解，导致自噬溶酶体中LC3含量降低[22]。在自噬活动中，Atg3、Atg7作为LC3泛素样偶联系统中重要组成成分，在液泡近端层次上募集，并组织成为对自噬体形成至关重要的前自噬体结构[23]。此外，P62作为连接LC3和聚泛素化蛋白之间的桥梁，被选择性地包裹进自噬体，之后被自噬溶酶体中的蛋白水解酶降解。在持续的应激状态及持续进展的自噬作用下，胞质中出现大量自噬溶酶体，降解胞内物质，导致细胞由于过度的自我损耗而死亡，即自噬性细胞死亡。本研究结果表明，ACY-738作用后，Atg3、Atg7、LC3、P62蛋白表达均下调，提示ACY-738可以诱导DLBCL细胞发生自噬性细胞死亡。

P53作为重要的抑癌基因，参与调控肿瘤细胞凋亡和细胞自噬等生理病理过程。当细胞处于DNA损伤或氧化应激时，P53通过磷酸化或乙酰化修饰激活，激活的P53蛋白参与多种基因的转录，如BAX、PUMA等，从而诱导细胞发生凋亡。此外，P53还可以促进肿瘤抑制因子PTEN的表达，进一步抑制mTOR的活性从而诱导自噬[24]–[25]。考虑到ACY-738为HDAC6的抑制剂，我们推测，ACY-738可能通过表观遗传调控DLBCL细胞凋亡和自噬。本研究发现ACY-738作用后，可以乙酰化激活P53，从而诱导细胞发生P53依赖的凋亡及自噬。

综上所述，本研究发现HDAC6抑制剂ACY-738可以调控P53介导的细胞凋亡和自噬，从而抑制DLBCL细胞增殖。但本研究尚有不足之处，未在动物体内进一步验证ACY-738的药效学评价，本研究初步明确了ACY-738有潜力成为抗DLBCL的靶向药物，但其分子机制仍需进行深入探索。
